# Microbiome and Antimicrobial Resistance Gene Dynamics in International Travelers

**DOI:** 10.3201/eid2507.181492

**Published:** 2019-07

**Authors:** Charles Langelier, Michael Graves, Katrina Kalantar, Saharai Caldera, Robert Durrant, Mark Fisher, Richard Backman, Windy Tanner, Joseph L. DeRisi, Daniel T. Leung

**Affiliations:** University of California, San Francisco, California, USA (C. Langelier, K. Kalantar, S. Caldera, J.L. DeRisi);; Chan Zuckerberg Biohub, San Francisco (C. Langelier, S. Caldera, J.L. DeRisi);; University of Utah School of Medicine, Salt Lake City, Utah, USA (M. Graves, R. Durrant, M. Fisher, R. Backman, W. Tanner, D.T. Leung);; ARUP Laboratories, Salt Lake City (R. Durrant, M. Fisher)

**Keywords:** microbiome, antimicrobial resistance, resistome, travel medicine, ESBL, extended-spectrum β-lactamase, Enterobacteriaceae, bacteria

## Abstract

We used metagenomic next-generation sequencing to longitudinally assess the gut microbiota and antimicrobial resistomes of international travelers to clarify global exchange of resistant organisms. Travel resulted in an increase in antimicrobial resistance genes and a greater proportion of *Escherichia* species within gut microbial communities without impacting diversity.

International travel is a known contributor to the emergence of organisms with antimicrobial resistance (AMR) (*1*–*4*). Colonization with resistant microbes acquired during travel can persist asymptomatically for extended periods and result in transmission into the environment and susceptible populations (*5*). The mechanisms underlying acquisition of AMR bacteria during travel are incompletely understood, although changes in the intestinal microbiota are hypothesized to play a role (*6*). To clarify AMR exchange during global travel, we used metagenomic next-generation sequencing (mNGS) to assess gut microbiota composition and the antimicrobial resistome.

## The Study

During March 2016–2018, we recruited adults with planned travel to Asia or Africa for healthcare-related work. Participants introduced 1 tablespoon of stool into vials with either RNAprotect (QIAGEN, http://www.qiagen.com) or Cary-Blair (CB) media and then submitted samples and surveys pretravel, posttravel, 30 days posttravel, and 6 months posttravel. Upon receipt, we stored RNAprotect samples at −80°C and CB samples at 4°C until inoculation onto chromogenic agar plates selective for extended-spectrum β-lactamase (ESBL)–producing bacteria (CHROMagar ESBL) and incubation overnight at 37°C. We then inoculated single colonies into LB broth and incubated overnight at 37°C. If multiple morphotypes were identified, we conducted separate subcultures. We performed speciation by using matrix-assisted laser desorption/ionization time-of-flight mass spectrometry.

DNA and RNA extracted using the QIAGEN Powerfecal kit underwent metagenomic sequencing as previously described (*7*). Raw data are available publicly (Bioproject PRJNA509512). We detected enteric microbiota using a recently developed bioinformatics pipeline (*7*). We aggregated microbial alignments at the genus level before downstream analyses. To control for background environmental and reagent contaminants, we incorporated no-template water control samples alongside extracted nucleic acid and carried them forward throughout library preparation and sequencing. We then performed direct subtraction of total reads aligning to each microbial genus present in controls from each study sample before downstream analyses.

We used the SRST2 computational tool and ARGannot_r2 database (https://github.com/katholt/srst2) to identify AMR genes with allele coverage of >20% (*8*). Although a precise definition of ESBL has not been established, we used a working definition of Ambler class A–D β-lactamases with known or predicted ability to confer resistance to first- through third-generation cephalosporins (*9**,**10*). We required detection of chromosomally encoded Ambler class C β-lactamases (i.e., AmpC) by both DNA-Seq and RNA-Seq to capture actively expressed genes.

Nine of 10 participants were culture-positive for ESBL-producing *Escherichia coli* (ESBL-PE) upon return, including 8 persons who traveled to Nepal and 1 who went to Nigeria. One traveler was found to be colonized before departure (traveler 3 [T3]); 3 travelers had persistent carriage at the 30-day visit (T2, T3, and T5) and 2 at 6 months (T3 and T5) (Table 1). Although 4 participants experienced diarrheal symptoms during travel, only 1 (T5) had persistent diarrheal symptoms at 6 months. Diarrheal symptoms were not associated with persistent ESBL-PE colonization at any point, and no travelers reported antibiotic use or receipt of healthcare in an inpatient setting while traveling. All travelers were exposed to inpatient healthcare facilities, and 3 travelers reported street food consumption.

**Table 1:** Selected characteristics of 10 travelers and assessment of ESBL-producing Enterobacteraciae*

Traveler	Destination	Duration, d	Diarrhea	Pretravel	Posttravel	30 d posttravel	6 mo posttravel
T1	Nepal	30	N	–	*AmpC*	*OXA-209*	*OXA-209*
T2	Nepal	30	N	–	*AmpC, CTX-M1*	*AmpC*	–
T3	Nepal	30	N	*OXA-209*	*AmpC, CTX-M1, OXA-209*	*OXA-209*	*OXA-209*
T4	Nepal	16	N	–	*AmpC*	–	NR
T5	Nepal	30	Y	–	*AmpC, SHV-12*	*AmpC*	*AmpC*, *CTX-M-1*
T6	Nepal	15	Y	–	*AmpC, CTX-M-1*	–	–
T7	Nepal	18	N	–	*AmpC, CTX-M-1*	NR	NR
T8	Uganda	14	N	–	– (*AmpC*)†	–	–
T9	Nigeria	60	Y	–	*AmpC*	–	–
T10	Nepal	30	Y	–	*AmpC, CTX-M-1*	–	NR

*Participants submitted samples pretravel (within 1 week before departure), posttravel (within 1 week after return), 30 days posttravel, and 6 months posttravel. ESBL, extended-spectrum β-lactamase; N, no; NR, no sample received; Y, yes; –, ESBL-negative by culture. †T8 was the only participant who was phenotypically ESBL-negative after travel.

We first examined changes in gut microbiome α diversity after international travel (Figure, panel A) and found that the Shannon diversity index (SDI) did not significantly change upon return (p = 0.674 by Wilcoxon rank-sum test) or at day 30 posttravel (p = 0.250 by Wilcoxon rank-sum test). We then assessed whether microbial community composition differed across all participants at their posttravel versus pretravel visit but found no difference (Bray Curtis Index p = 0.23 by permutational multivariate analysis of variance). Although global composition and diversity of gut microbiota did not significantly change after travel, we observed significant differences in the abundance of discrete genera. Across all participants, *Enterobacteriaceae* demonstrated the greatest fold change in abundance posttravel; *Escherichia* was the genus most differentially increased (p<0.001 by Wilcoxon rank-sum test) (Figure, panel B; Appendix Figures 1, 2). 

**Figure:**
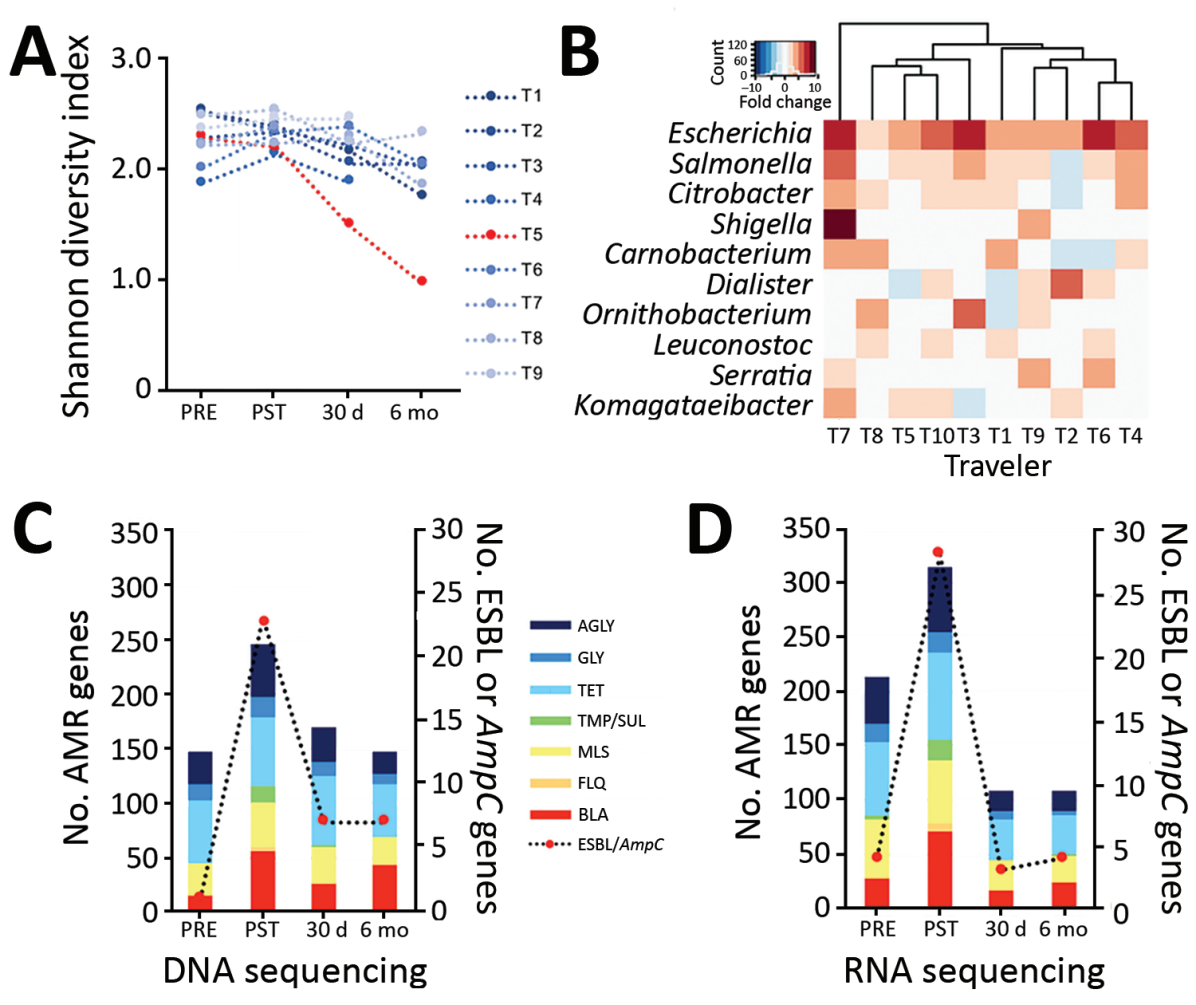
Microbiome and AMR gene dynamics in international travelers. A) Longitudinal profile of traveler gut microbiome diversity measured by Shannon diversity Index. Traveler 5 (T5) had a Shannon diversity index >3 SDs below the mean when measured at 30 days and 6 months posttravel. B) Microbes, by genus, demonstrating the greatest fold change in abundance after travel on the basis of DNA sequencing nucleotide alignments. C) Total number of AMR genes identified with >20% allele coverage by DNA sequencing. D) Total number of AMR genes identified with >20% allele coverage by RNA sequencing. AGLY, aminoglycoside; AMR, antimicrobial resistance; BLA, β-lactamase; FLQ, fluoroquinolone; MLS, macrolide, lincosamide, streptogramin; PRE, pretravel (within 1 week before departure); PST, posttravel (within 1 week after return); TMP/SUL, trimethoprim/sulfamethoxazole; TET, tetracycline; ESBL, extended-spectrum β-lactamase.

Analysis of the antimicrobial resistome revealed an increase in AMR genes and transcripts after return from travel (p<0.01 for DNA and p = 0.03 for RNA sequencing, both by Wilcoxon rank-sum test) (Table 2; Figure, panels C–D; Appendix Figure 3). ESBL-encoding genes, *AmpC*-encoding genes, or both were identified in 100% of samples with an ESBL culture-determined phenotype and in 14% of samples without, including 1 participant (T8) who was phenotypically ESBL-negative after travel (Table 1). β-lactam–resistance genes increased posttravel, including *AmpC*, *CTX-M*, *OXA*, and *SHV* gene families known or predicted to confer ESBL production, as well as diverse additional β- lactamase genes (Appendix Figure 3). Travel also resulted in an increase in *qnr* plasmid–mediated quinolone-resistance genes, as well as trimethoprim (*dfr*)-, sulfa-, macrolide-, and aminoglycoside-resistance genes (Table 2). Genes conferring resistance to tetracyclines and aminoglycosides were most abundant in travelers at baseline and remained stable or decreased during travel (Table 2).

**Table 2:** Fold change in abundance of AMR genes found in *Enterobacteraciae* with >20% allele coverage compared with pretravel, by resistance gene or drug class*

Resistance gene or drug class	Fold change compared with pretravel							
	Posttravel			30 d			6 mo	
	DNA	RNA		DNA	RNA		DNA	RNA
β*-*lactam AMR genes								
* AmpC*	>100	>100		>100	1		>100	2
* AmpH*	>100	>100		>100	2		>100	1
* CTX*	>100	>100		>100	>100		>100	>100
* MrdA*	50	>100		0	>100		5	>100
* OXA*	2	1		1	0		1	0
* SHV*	>100	>100		>100	>100		>100	>100
* TEM*	>100	64		54	0		15	1
Other antibiotic classes								
Aminoglycoside	2	3		1	0		0	0
Fluoroquinolone	>100	>100		>100	>100		>100	>100
Glycopeptide	0	1		0	0		0	0
Macrolide, lincosamide, streptogramin	2	3		1	3		1	1
Sulfa	22	29		1	0		0	0
Tetracycline	1	1		1	0		1	0
Trimethoprim	>100	88		>100	0		>100	1

*Among 10 participants who submitted samples pretravel (within 1 week before departure), posttravel (within 1 week after return), 30 days posttravel, and 6 month posttravel. AMR, antimicrobial resistance.

We found no significant differences in SDI between persistent carriers and those who lost carriage at 30 days (n = 3; p = 0.56 by *t*-test) or at 6 months (n = 2; p = 0.27 by *t*-test) posttravel. T5, who was colonized at both time points and who was the only traveler with persistent diarrheal symptoms, had an SDI >3 SDs below the mean at 6 months (Figure, panel A). Bray-Curtis distance measured pretravel or posttravel did not differ between travelers who were ESBL-PE positive by culture at 30 days or 6 months posttravel (p = 0.32 by permutational multivariate analysis of variance). No individual taxa were associated with posttravel ESBL positivity on the basis of an adjusted p value <0.05 (*t*-test) at 30 days or 6 months posttravel.

## Conclusions

International travel is a well-recognized contributor to the global spread of emerging infectious diseases, including AMR bacteria (*1*,*4*). We analyzed the enteric microbiota and resistomes of returned travelers and found a marked increase in AMR genes that was associated with an increased proportion of *Escherichia* bacteria. At baseline, few participants had evidence of ESBL colonization; however, after travel, ESBL and actively transcribed *AmpC* genes were notably increased, consistent with previous reports (*4*,*11*). Both mNGS and culture-based methods found evidence of persistent ESBL colonization after 6 months, suggesting that travel can induce long-term changes in the antimicrobial resistome (*5*). In addition to β-lactamase genes, mNGS identified a diversity of other AMR gene classes that increased in abundance after travel. For example, 80% of participants acquired horizontally transferable *qnr* genes after travel, reflecting the limited utility of quinolones for treatment of traveler’s diarrhea (*12*). Although we did not detect genes known to encode carbapenemases, participants might have harbored carbapenem-resistant *Enterobacteraciae*, given that a combination of an ESBL or *AmpC* gene with a porin mutation or efflux pump can lead to carbapenem resistance (*13*).

Changes in microbiome diversity were not associated with ESBL positivity at 30 days or 6 months posttravel, suggesting that disruption of the antimicrobial resistome can occur in the setting of a preserved microbial community structure. We observed a high rate of ESBL-PE acquisition in this cohort, most of whom traveled to the Indian subcontinent, consistent with previous studies of travelers returning from this region (*1*). Notably, none of the travelers in this cohort reported antibiotic use, suggesting that substantial ESBL-PE colonization can occur even in the absence of antibiotic-related disruption of commensal gut microbiota.

Because this study is limited by small sample size, relevant associations might have been missed. In addition, we could not assess the presence of carbapenem-resistant *Enterobacteriaceae* by using culture-based methods. mNGS and phenotypic antimicrobial resistance need assessment in larger cohorts traveling to more destinations. Nonetheless, our findings highlight the pervasiveness of AMR microbe exchange during international travel and the promise of mNGS for assessing the global exchange of antimicrobial resistance.

Appendix Additional information on microbiome and antimicrobial resistance gene dynamics in international travelers.
