# Supplementary material for: Microbiome and Antimicrobial Resistance Gene Dynamics in International Travelers
Source: Emerg Infect Dis. 2019 Jul;25(7):1380–3. doi: 10.3201/eid2507.181492 (PMC6590773; doi:10.3201/eid2507.181492)
Supplement: Appendix — Additional information on microbiome and antimicrobial resistance gene dynamics in international travelers. [file 18-1492-Techapp-s1.pdf]

Article DOI: https://doi.org/10.3201/eid2507.181492

# Microbiome and Antimicrobial Resistance Gene Dynamics in International Travelers

## Appendix

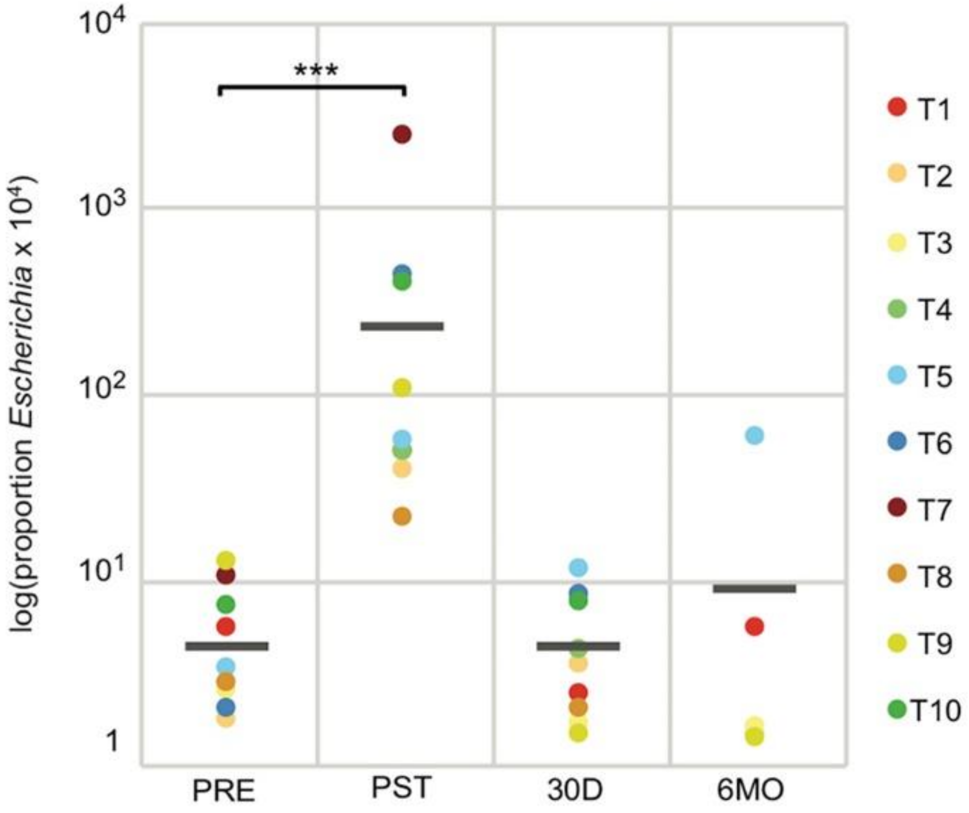


**Appendix Figure 1.** Assessment of relative abundance of *Escherichia* over time, pre- and post-travel, revealed an increase ($p<0.001$ by Wilcoxon Rank Sum) post-travel.

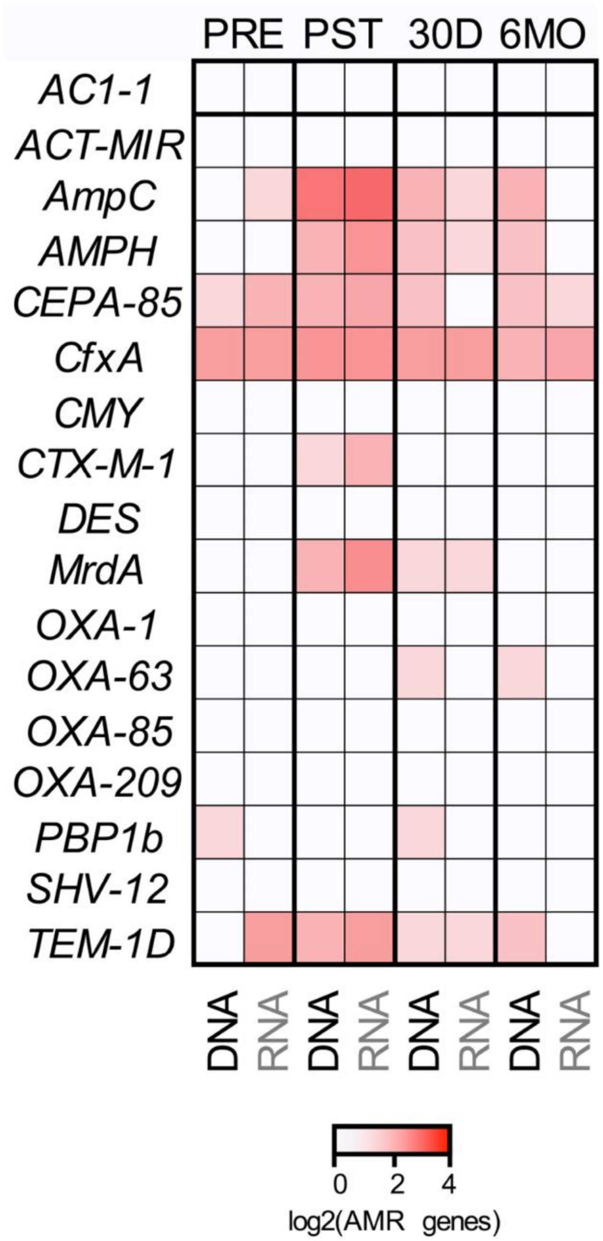


**Appendix Figure 2.** Relative proportion of the 20 most abundant microbial genera present in the enteric microbiomes of international travelers, assessed longitudinally using shotgun metagenomic DNA sequencing. Participants submitted samples pre-travel (PRE, within 1 week before departure), post-travel (PST, within 1 week after return), 30 days post-travel (30D), and 6 months post-travel (6MO).

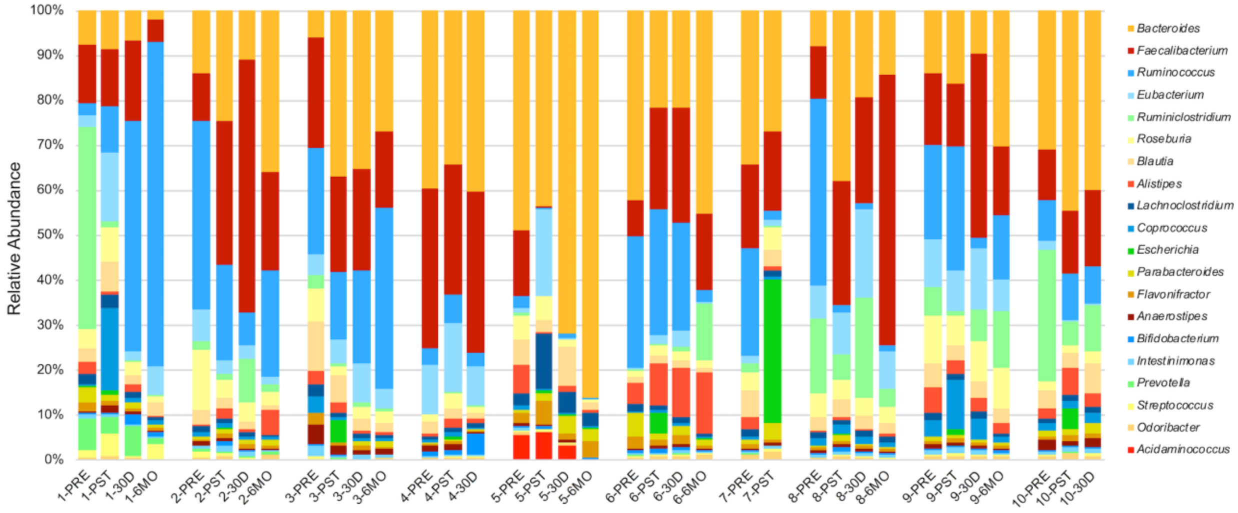


**Appendix Figure** 3**.** Beta-lactamase genes identified in the gut microbiomes of travelers over time, pre- and post-travel. Gradient shading corresponds to gene abundance (average allele sequencing depth per million reads mapped, AdpM).
